# Detecting and Visualizing Outliers in Provider Profiling Using Funnel Plots and Mixed Effects Models—An Example from Prescription Claims Data

**DOI:** 10.3390/ijerph15092015

**Published:** 2018-09-15

**Authors:** Oliver Hirsch, Norbert Donner-Banzhoff, Maike Schulz, Michael Erhart

**Affiliations:** 1 Department of General Practice/Family Medicine, Philipps University Marburg, Karl-von-Frisch-Str.4, 35043 Marburg, Germany; norbert@staff.uni-marburg.de; 2 Central Research Institute of Ambulatory Health Care in Germany (ZI), Salzufer 8, 10587 Berlin, Germany; mschulz@zi.de (M.S.); merhart@zi.de (M.E.)

**Keywords:** drug prescriptions, ambulatory care, statistical data interpretation, reference standards

## Abstract

When prescribing a drug for a patient, a physician also has to consider economic aspects. We were interested in the feasibility and validity of profiling based on funnel plots and mixed effect models for the surveillance of German ambulatory care physicians’ prescribing. We analyzed prescriptions issued to patients with a health insurance card attending neurologists’ and psychiatrists’ ambulatory practices in the German federal state of Saarland. The German National Association of Statutory Health Insurance Physicians developed a prescribing assessment scheme (PAS) which contains a systematic appraisal of the benefit of drugs for so far 12 different indications. The drugs have been classified on the basis of their clinical evidence as “standard”, “reserve” or “third level” medication. We had 152.583 prescriptions in 56 practices available for analysis. A total of 38.796 patients received these prescriptions. The funnel plot approach with additive correction for overdispersion was almost equivalent to a mixed effects model which directly took the multilevel structure of the data into account. In the first case three practices were labeled as outliers, the mixed effects model resulted in two outliers. We suggest that both techniques should be routinely applied within a surveillance system of prescription claims data.

## 1. Introduction

When prescribing a drug for a patient, a physician not only has to consider the individual needs of a patient, the severity of her/his condition, the characteristics of suitable drugs and potential interactions with simultaneously prescribed treatments, but also economic aspects. For many indications several treatment options exist. Among these, prescribing physicians are expected to choose the less costly ones in order to preserve the economic viability of the health care system. However, the success of treatments should not be compromised. In other words, prescribers are expected to balance quality and costs when prescribing treatments for their patients. In the German health care system this is a requirement based on federal law [1].

In Germany, prescriptions for drugs issued by physicians in the ambulatory care sector are centrally recorded and surveyed regularly. Analytical procedures focus on prescribing costs of individual physicians and/or practices. A certain deviation from the average of a specialty leads to investigations with regard to the appropriateness of prescriptions. If high costs cannot be explained and justified by case-mix of patients, prescribers have to undergo remedial procedures and risk even financial sanctions. These procedures have come under criticisms recently. Patients are being referred for high-cost prescriptions only which create additional costs and render coordination of care more complex. Primary care physicians providing comprehensive care, including the prescription of costly medications, are being punished, to name but two objections against the established procedure.

The German National Association of Statutory Health Insurance Physicians has developed a prescribing assessment scheme (PAS) which contains a systematic appraisal of the benefit and cost effectiveness of important drugs [2]. It is based on published clinical trials, systematic reviews, guidelines and appraisals by institutions such as the National Institute for Health and Care Excellence (NICE) or the Institute for Quality and Efficiency in Health Care (IQWIG). The following clinical areas have been covered so far: hypertension, coronary heart disease, lipid disorders, osteoporosis, chronic heart failure, atrial fibrillation, Alzheimer dementia, depression, diabetes mellitus type 2, upper and lower respiratory tract infections, urinary tract infection, asthma, and chronic obstructive pulmonary disease (COPD). Drugs for these conditions have been classified on the basis of their clinical evidence as “standard”, “reserve”, or “third level” medication. In the Results section, we present analyses regarding the appropriateness of prescriptions based on the PAS.

The identification of providers issuing a proportion of inappropriate prescriptions regarded as too high is essentially a case of profiling. Profiling is defined as the identification of unusual provider performance (hospitals, departments, practices, physicians) compared to other providers or to a reference standard [3,4]. Previously published profiling analyses were conducted at the patient, individual clinician, and organizational level [3]. Processes, results (clinical outcomes), and costs were investigated. Hospital mortality is perhaps the aspect having gained most attention in the past [5,6].

While explicit policies, e.g., treatment pathways or local guidelines, or implicit policies, e.g., not well-defined thresholds for referral or prescription of antibiotics, are the main focus of profiling procedures, there are other sources of variation in the data analyzed in this way. Apart from random variation, patient characteristics before treatment are particularly relevant [7]. Therefore, it is important to adjust for case-mix which includes factors like age, gender, presence of disease(s) and severity of disease. Within the profiling paradigm, funnel plots have become the preferred graphical means to assess the performance of healthcare providers [8]. Recently, this approach was applied in order to identify inappropriate prescribing patterns [9,10,11,12], but few studies explicitly used statistical techniques accounting for the multilevel structure of the data [13,14].

We were interested in the feasibility and validity of profiling based on funnel plots for the surveillance of ambulatory care physicians’ prescribing. For this analysis we focus on drugs prescribed by psychiatrists and neurologists in the Saarland, one of the 16 German federal states combining both urban and rural characteristics.

## 2. Materials and Methods

The description of methods follows the recently published Reporting of studies Conducted using Observational Routinely-collected Data (RECORD) statement [15].

### 2.1. Data

We analyzed prescription data of 2014 according to § 300 Section 2, Social Security Code V (AVD). These data were based on patients with a health insurance card attending neurologists’ and psychiatrists’ ambulatory practices. Virtually all of these had contracts with the statutory health insurance system in the German federal state of Saarland. Since neurologists and psychiatrists could only be identified in the data set if they practiced on their own, multispecialty practices were not included in the analyses presented here. In order to exclude artefacts and practices providing predominantly psychotherapy, we selected only practices with at least five prescriptions per quarter. These were prescriptions covered by an evidence based prescribing assessment scheme (PAS) which were filled by patients of the statutory health insurance system in pharmacies of their choice. The level of analysis was the individual prescription. A total of 56 (67.5%) out of 83 practices fulfilled the inclusion criteria for our analyses.

Patient subjects were constructed by prescription records with the same health insurance card number plus same health insurance institution number and same birthday. This information was stored on the health insurance card and was read every time a patient received a prescription. Before and after patient-construction these variables were pseudonymized by a hashing-function including a secret. Each prescription record contained the prescribing physician’s individual identification number as well as his/her practice identification. Both variables were pseudonymized by hashing-function including a secret.

The patient-prescription records were linked with patients’ diagnoses taken from the administrative claims data of 2014 according to § 299, Social Security Code V (VDA). A deterministic record-linkage was performed at the patient-level using the patient-pseudonym issued from health insurance card number plus health insurance institution number plus birthday issued from both AVD and VDA data.

Variables under study were patients’ age and gender, the total number of different drugs per patient prescribed from all health physicians the patient had contact with. An index of patient need/risk for health services utilization was calculated according to the Patient classification system of the Institut des Bewertungsausschusses (INBA). This index was based on the regression of ambulatory health insurance costs on 34 Age- and gender groups and 72 disease-categories such as diabetes, HIV/AIDS, ophthalmologic-diseases, etc. Resulting from the regression analyses performed by INBA, each patient was assigned his or her expected ambulatory health insurance cost. The latter value constitutes the patient risk score used in this study. This was essentially a measure of morbidity and health service needs [16].

The German National Association of Statutory Health Insurance Physicians developed a prescribing assessment scheme (PAS) which contains a systematic appraisal of the benefit and cost effectiveness of important drugs [2]. The drugs have been classified on the basis of their clinical evidence as “standard”, “reserve” or “third level” medication. The main objective of the PAS is to give providers feedback on their prescribing. In the Results section, we present analyses referring to all prescriptions covered by the PAS. We dichotomized the three-level PAS variable to “third level” versus “standard or reserve”.

### 2.2. Statistical Methods

Funnel plots have become the preferred graphical means to assess the performance of healthcare providers within the profiling paradigm. They consist of four components [8]:

- An indicator: this is the outcome of interest, plotted on the y-axis.
- A target: this is the normative expectation for the respective population which is often operationalized by the sample mean or by other measures of central tendency. An alternative would be an external goal (e.g., certain number of infections with multiresistent germs per 1000 patients; certain proportion of prescription of non-evidence-based medication).
- A precision parameter: this parameter determines the accuracy with which the indicator is being measured. Precision is connected to the variance of the outcome and the number of observations, such as total number of patients or total number of prescriptions of non-evidence-based medication.
- Control limits: these are the limits beyond which a provider would be classified as showing unusual performance. They are often set to a probability of 5% and 0.2% for a provider truly within the control limits to be classified as showing unusual behavior and thus constituting a false positive result [8]. Thus, even if a provider truly is showing usual behavior (on average) there is variation in his/her behavior. This variation could be attributable to so called unmeasured heterogeneity and is labelled as variation due to “chance”. Picking up hypothetically 1000 providers with truly usual behavior, a 5% limit for example separates the 25 providers with the most positive behavior on the one end and the 25 providers with the most negative behavior on the other end. These are the 5% control limits meaning that 50 out of the 1000 hypothetical providers will be falsely classified as unusual.

However, how to define and measure a deviation from the norm is controversial [17]. The interval of adequate performance should preferably be based on considerations regarding clinical content. Often, however, this poses considerable difficulties and purely statistical considerations prevail [5].

Often an unconvincingly large proportion of providers falls outside the prespecified control limits. This may be due to “overdispersion” which results from not all relevant covariates being taken into account [18]. Procedures have been suggested to adjust for this [19]. Factors contributing to excessive variability causing overdispersion may operate at the individual, e.g., patient, or the aggregate or cluster level, e.g., provider, department or hospital [20].

Profiling and other statistical methods trying to identify divergent providers face a fundamental paradox. On the basis of available data, a statistical model (distribution) has to be defined. Deviating units, which the procedure is meant to detect, are part of the sample providing the data. The more these divergent entities are incorporated into the statistical model the less likely investigators or administrators will be able to detect them [21]. As a consequence, one should select robust models that are less affected by idiosyncrasies contained in the data available for analysis [22].

We followed the approach of detecting and visualizing outliers in provider profiling via funnel plots and mixed effect models demonstrated by Ieva and Paganoni [23]. This approach implies analyses on an individual and an aggregate level. The statistical unit of the individual level 1 is the prescription of a drug. The statistical level on the aggregate level is the practice.

In a first step we plotted the number of prescriptions covered by the PAS against the proportion of medication with “third-level”. We then calculated a common mean model with the R procedure glm and level 1 predictors patient age, -gender, -individual morbidity without age and gender effects, and individual polypharmacy ≥2 quarters to predict the occurrence of a third level medication (yes/no). These predictors were selected on the basis of procedures glmer of R package lme4 and glmmPQL of R package nlme.

Next, we calculated the observed versus the expected ratio of third level medication for each practice and plotted it against the expected number of third level medication in an unadjusted funnel plot [23]. Practices above the upper control limits of the funnel plot can be regarded as outliers, i.e., in need of measures to have their prescribing habits explored and possibly improved. In a next step, the Wϕ^ was calculated according to the method of Spiegelhalter [8] to verify if overdispersion was present. We then applied the additive adjustment factor for the band limits of the funnel plot described in [23] and demonstrated the change in the number of outliers in an adjusted funnel plot. To validate the funnel plot procedure, we fitted a mixed effect model which accounted for the cluster structure of the data with the R procedure glmer and predictors age, gender, individual morbidity as described above, and individual polypharmacy ≥2 quarters as fixed effects and a random intercept.

A particular patient could receive several prescriptions. Thus, the individual prescriptions were not statistically independent but were clustered at the patient level. Furthermore, one patient could receive prescriptions from several physicians. Thus, from a strict statistical perspective a 3-level hierarchical model accounting for cross-classification should be employed. However, approximately 150 thousand prescriptions nested in approximately 39 thousand patients nested in 57 practices would demand a sizeable computational capacity. For the sake of presenting a feasible and already tested analytic strategy, we therefore decided to follow the approach of detecting and visualizing outliers in provider profiling via funnel plots and mixed effect models demonstrated by Ieva and Paganoni [23] while at the same time acknowledging that we might have underestimated complexity and variation of the data.

A boxplot and a Q-Q plot of the point estimates of the random intercept of the multilevel model were drawn to assess the agreement between the two approaches. We performed all calculations with R, Version 3.2.2 (https://www.r-project.org/).

We obtained a waiver by the local ethics committee of the Department of Medicine at Phillips University Marburg for this study of fully anonymous prescription claims data, dated 9 December 2015, project identification code: “Az.: Prof. Ri./ra”.

## 3. Results

**Figure 1:**
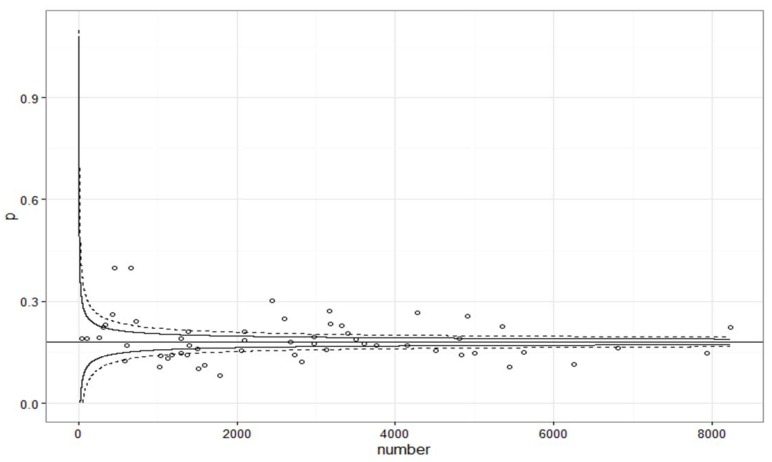
Number of prescriptions by neurologists and psychiatrists in the German federal state of Saarland plotted against the proportion of medication with third level. Practices are units of observation. Control limits were calculated according to Ieva and Paganoni and Spiegelhalter [8,23]. The solid horizontal line represents Huber’s M estimator, a robust substitute for the mean.

Our data set contained 155,234 prescriptions issued by 56 practices. After elimination of missing data in age, gender, prescription or diagnosis variables 152,583 prescriptions remained. A total of 38,796 patients received these prescriptions. Mean age was 59.2 years, 25% were younger than 49 years, 25% were older than 73 years. A proportion of 63.6% were female. On average, every patient received 3.9 prescriptions from a psychiatrist/neurologist. About 46.1% of the patients received 6 or more agents on at least two quarters and thus were classified as polypharmacy patients. On average the patients had a risk score of 1417. This means that their expected use of ambulatory health care-services was about 41.7% higher than the average of patients. We first plotted the total number of prescriptions against the proportion of medication with third level which is displayed in Figure 1.

As is apparent in Figure 1, about one fourth of practices above the upper control limits of the funnel plot would be categorized as outliers. Given the size of this group, overdispersion is likely to contribute to these findings [8,23].

**Figure 2:**
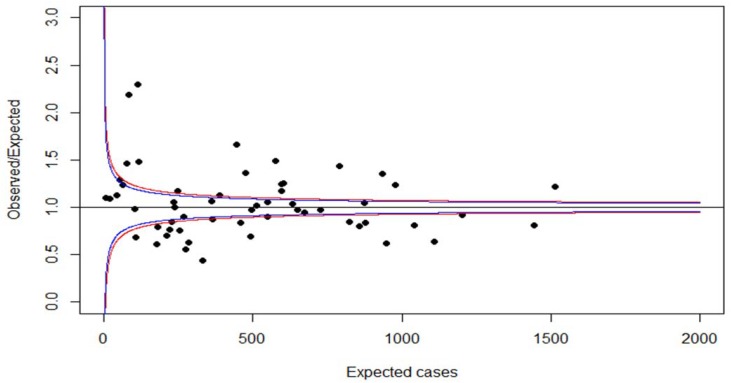
Standardized ratio of third level medication against expected number of third level medication in neurologists and psychiatrists in the German federal state of Saarland. Adjusted funnel plot with band limits at 95% (blue lines) and 98% (red lines).

We then calculated a common mean model with the R procedure glm and level 1 predictors age, gender, individual morbidity, and individual polypharmacy ≥2 quarters to predict the occurrence of a third level medication (yes/no). Age (*p* < 0.001), gender (*p* < 0.001), individual morbidity and individual polypharmacy ≥2 quarters (*p* < 0.001), were significant predictors. Next, the observed versus the expected ratio of third level medication was calculated for each practice and plotted against the expected number of third level medication (see Figure 2) which explains the difference to the numbers on the x-axis of Figure 1.

Again, about one fourth of practices would be classified as outliers. So far, we did not consider overdispersion. In our case, the Wϕ^=32.01 which signals overdispersion as it should not be higher than Wϕ^ = 1 + 2$\sqrt{2/56}=$ 1.38 [23].

**Figure 3:**
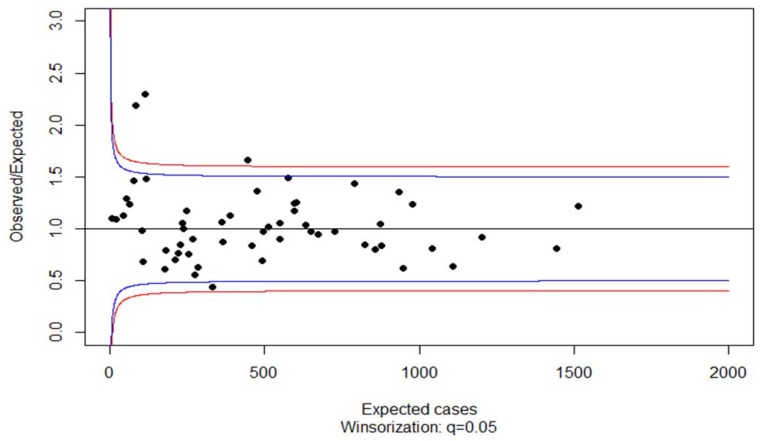
Standardized ratio of third level medication in neurologists and psychiatrists in the German federal state of Saarland. Additive adjusted funnel plot with band limits at 95% (blue lines) and 98% (red lines).

Consequently, we applied the additive adjustment factor for the band limits of the funnel plot. The resulting funnel plot is displayed in Figure 3.

The funnel plot in Figure 3 classifies only three practices as having an excessive proportion of third level prescriptions. In order to validate the funnel plot procedure for detecting outliers we applied the method of identifying extreme values of the random effect point estimates from a mixed effect model. We fitted a mixed effect model with the R procedure glmer with the former covariates as fixed effects and a random intercept. This time accounting for the cluster structure of the data, age, gender, and individual morbidity without age and gender effects (all *p* < 0.001) and individual polypharmacy (*p* = 0.003) were significant predictors of a third level prescription at the patient level, with odds ratios of age 0.9912, female gender 0.7938, morbidity 1.2362, and polypharmacy 1.0487. Results hinting at males and patients suffering from severe illness are more likely to receive third level medication. However, the actual effects are rather small indicating that the appropriateness of prescription depends only to a very small degree on the variables explored in our data set.

**Figure 4:**
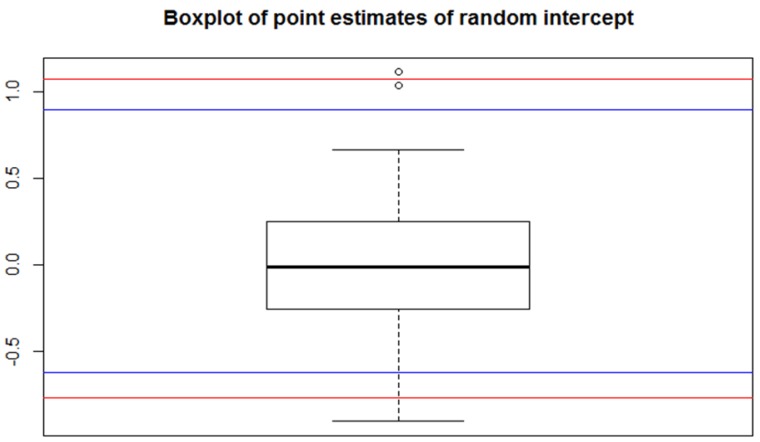
Boxplot of the point estimates of the random intercept of the multilevel model. The horizontal blue lines correspond to the quantiles of 2.5% and 97.5%. The horizontal red lines correspond to the quantiles of 1% and 99%.

The two points over the limit of the whiskers in the boxplot of the point estimates of the random intercept (Figure 4) correspond to two of the three practices that were out of the 95% and 98% band limits of the funnel plot in Figure 3.

**Figure 5:**
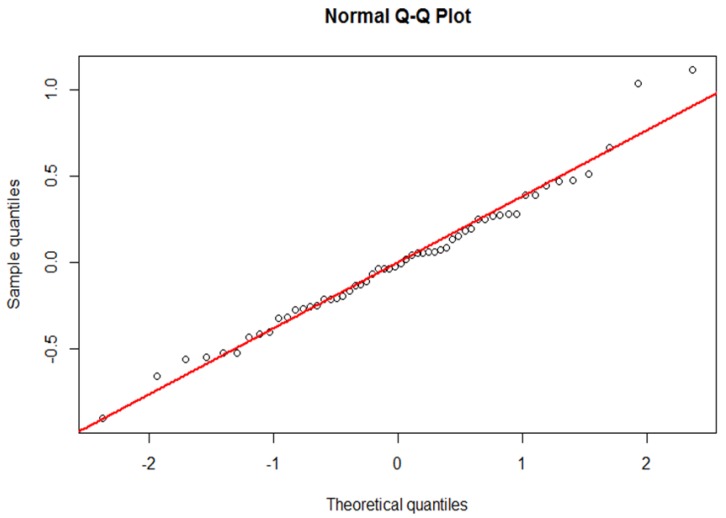
Normal Q-Q plot of the point estimates of the random intercept of the multilevel model to visualize outliers.

These two practices can further be located in the upper left part of the Normal Q-Q plot in Figure 5. We conclude that funnel plots and mixed effect models reached almost the same conclusions in detecting outliers in German prescription claims data.

## 4. Discussion

To our knowledge this is the first time the method for detecting and visualizing outliers in provider profiling using funnel plots and mixed effects models [23] has been applied to prescription claims data in Germany. Prescriptions issued by neurologists and psychiatrists in the German federal state of Saarland were evaluated by an appropriateness of prescribing index. The funnel plot approach with additive correction for overdispersion was almost equivalent to a mixed effects model which directly took the multilevel structure of the data into account. In the first case three practices were labeled as outliers, the mixed effects model resulted in two, which were both among the practices identified by the first model. In our study, morbidity and polypharmacy as significant predictors corroborate earlier findings showing considerable associations to high risk prescribing [14].

Although results of funnel plot and mixed effect model profiling were similar in our case, we suggest that both techniques should be routinely applied in profiling of prescription behavior. The prescription behavior of individual physicians is usually highly consistent across their respective patients but different from other physicians of their discipline. Physician-level clustering should therefore be accommodated for [24].

For decades it has been recognized that claims-based profiling methods can be applied to improve the quality of care [25]. The focus, however, should be more on quality than on the cost of care as evidence-based treatments are more likely to reduce symptom load in patients and should therefore have priority.

Profiling can be applied in a formative, non-punitive sense. Providers can thus identify areas of problematic actions and draw conclusions for improvements. Information from profiling analyses can be integrated into clinical pathways and treatment protocols in order to improve the provision of healthcare [3]. One of the primary goals of provider profiling in the area of prescription data should be to provide physicians with meaningful feedback regarding their behavior. If this is not in accordance with common guidelines or a certain target behavior, quality-oriented audits may help to elucidate the processes behind possible unusual results [18]. It is important to stress that the profiling approach discussed in this article primarily focuses on quality of prescriptions [25]. We are in line with other studies showing that practices with high prescribing costs per patient do not necessarily have prescription patterns which have to be modified. This might be due to special patient characteristics [12].

Some issues should be considered in connection with profiling. For example, statistical models seek to reduce information inherent in data which are extracted from observations of a group of entities. The aim is to represent the data as good as possible (“goodness of fit”). Such models are not suitable to draw conclusions about cases that were not in the calibration sample (“bias-variance-dilemma”). Therefore, more simple robust models should be preferred.

Funnel plots are an attractive tool for the surveillance physicians’ prescriptions. Within such a system there could be a continuous, systematic collection, analysis, and interpretation of prescription data used to inform health policy and planning [18] based on sound statistical methods and incorporating special provider characteristics. They are superior to simple rank-based methods like league tables as they are able to adjust for confounding variables. They can easily be created by common statistical software like R but trained personnel is needed to adequately interpret the results.

Past studies have shown equivocal results of feedback on prescribing behavior [26,27,28]. However, feedback was given delayed and irregularly. Immediate electronic feedback while prescriptions are issued should be the preferred method. This might be done best by providing electronic support during the prescription process. Primary care physicians who received feedback by a computerized decision support system (CDSS) considerably changed their prescription behavior compared to a control group [29]. This was confirmed in another study in which simple data feedback led to a significant reduction in high risk prescribing in primary care [30].

As a method to identify third-level-drugs by psychiatrists and neurologists in Saarland, the funnel method was feasible and largely concordant with a mixed effect model.

### Limitations

Prescription data was available for drugs that were completely reimbursed by the statutory health insurances in Germany. No data were available for over-the-counter (OTC) drugs, drugs prescribed for hospital in-patients or drugs reimbursed by private health insurances (<10% market share in the area covered). We were not able to evaluate the appropriateness of prescriptions in the individual clinical case. We had only limited information on patient characteristics and almost none on provider characteristics. This was due to logistical as well as formal reasons, such as privacy regulations. As a result, possibilities to adjust for confounders at these levels were limited [31,32]. Since neurologists and psychiatrists could be only identified in the data set if they practiced on their own, multispecialty practices were not included in the analyses presented here.

We chose the Saarland as the smallest German territorial federal state for practical and logistical reasons. Moreover, its proportion of rural and urban areas is largely representative for the whole country. This is confirmed by data from comparisons with other German federal states. The success index of Saarland which consists of parameters like economic growth, social security, unemployment rate, per capita gross national product, inner security, and gainful employment was with 5.86 close to the average in Germany of 5.56. The activity index of Saarland which contains factors that are under the influence of the German federal states and have interactions with the above-mentioned parameters was also close to the German average (5.18 versus 5.70, respectively) [33,34].

Although we could show the concordance between the funnel plot method and a mixed effects model, a true validity study would have required the individual investigation of practices and their prescribing behaviors which would have been beyond the scope of this project.

## 5. Conclusions

The prescribing assessment scheme (PAS) of the German National Association of Statutory Health Insurance Physicians is an instrument with which quality and cost-effectiveness of prescriptions can be assessed on the basis of evidence-based criteria. In this it is superior to costs, which have been used to evaluate physicians’ prescriptions in the past. The results of our study support the feasibility and validity of a funnel-plot approach together with mixed effect models.

## References

[1] Gemeinsamer Bundesausschuss (2016). Richtlinie des Gemeinsamen Bundesausschusses über die Verordnung von Arzneimitteln in der Vertragsärztlichen Versorgung [Guideline of the Common Federal Committee on the Prescription of Drugs in Ambulatory Care].

[2] Schwenzer S. (2015). ARMIN in Sachsen und Thüringen—Mehr Arzneimitteltherapiesicherheit durch rationale und evidenzbasierte Arzneimitteltherapie und patientenindividuelles Medikationsmanagement. Arzneiverordnung Praxis.

[3] Tucker J.L. (2000). The theory and methodology of provider profiling. Int. J. Health Care Qual. Assur..

[4] Gomes M., Gutacker N., Bojke C., Street A. (2016). Addressing Missing Data in Patient-Reported Outcome Measures (PROMS): Implications for the Use of PROMS for Comparing Provider Performance. Health Econ..

[5] Burgess J.F., Christiansen C.L., Michalak S.E., Morris C.N. (2000). Medical profiling: Improving standards and risk adjustments using hierarchical models. J. Health Econ..

[6] Bragg F., Cromwell D.A., Edozien L.C., Gurol-Urganci I., Mahmood T.A., Templeton A., van der Meulen J.H. (2010). Variation in rates of caesarean section among English NHS trusts after accounting for maternal and clinical risk: Cross sectional study. BMJ Clin. Res..

[7] Marshall E.C., Spiegelhalter D.J., Leyland A.H., Goldstein H. (2001). Institutional Performance. Multilevel Modelling of Health Statistics.

[8] Spiegelhalter D.J. (2005). Funnel plots for comparing institutional performance. Stat. Med..

[9] Pouwels K.B., Dolk F.C.K., Smith D.R.M., Robotham J.V., Smieszek T. (2018). Actual versus ‘ideal’ antibiotic prescribing for common conditions in English primary care. J. Antimicrob. Chemother..

[10] Hawker J.I., Smith S., Smith G.E., Morbey R., Johnson A.P., Fleming D.M., Shallcross L., Hayward A.C. (2014). Trends in antibiotic prescribing in primary care for clinical syndromes subject to national recommendations to reduce antibiotic resistance, UK 1995–2011: Analysis of a large database of primary care consultations. J. Antimicrob. Chemother..

[11] Gharbi M., Doerholt K., Vergnano S., Bielicki J.A., Paulus S., Menson E., Riordan A., Lyall H., Patel S.V., Bernatoniene J. (2016). Using a simple point-prevalence survey to define appropriate antibiotic prescribing in hospitalised children across the UK. BMJ Open.

[12] Tomlin A.M., Gillies T.D., Tilyard M.W., Dovey S.M. (2016). Variation in the pharmaceutical costs of New Zealand general practices: A national database linkage study. J. Public Health.

[13] Cahir C., Fahey T., Teljeur C., Bennett K. (2014). Prescriber variation in potentially inappropriate prescribing in older populations in Ireland. BMC Fam. Pract..

[14] Byrne C.J., Cahir C., Curran C., Bennett K. (2017). High-risk prescribing in an Irish primary care population: Trends and variation. Br. J. Clin. Pharmacol..

[15] Benchimol E.I., Smeeth L., Guttmann A., Harron K., Moher D., Petersen I., Sorensen H.T., von Elm E., Langan S.M. (2015). The REporting of studies Conducted using Observational Routinely-collected health Data (RECORD) statement. PLoS Med..

[16] Berger I., Hinz A., Naftalieva B., Schwan M., Spillner A. (2016). Bericht des Instituts des Bewertungsausschusses zur Weiterentwicklung des Klassifikationssystems sowie zur Ermittlung der Veränderungsraten für das Jahr 2015 Gemäß § 87a Abs. 5 SGB V. https://institut-ba.de/publikationen/InBA_Bericht_KM87a2014.pdf.

[17] Christiansen C.L., Morris C.N. (1997). Improving the Statistical Approach to Health Care Provider Profiling. Ann. Intern. Med..

[18] Dover D.C., Schopflocher D.P. (2011). Using funnel plots in public health surveillance. Popul. Health Metr..

[19] Cousineau D., Chartier S. (2010). Outliers detection and treatment: A review. Int. J. Psychol. Res..

[20] Spiegelhalter D.J. (2005). Handling over-dispersion of performance indicators. Qual. Saf. Health Care.

[21] Ohlssen D.I., Sharples L.D., Spiegelhalter D.J. (2007). A hierarchical modelling framework for identifying unusual performance in health care providers. J. R. Stat. Soc. Ser. A.

[22] Rousseeuw P.J., Leroy A.M. (2003). Robust Regression and Outlier Detection.

[23] Ieva F., Paganoni A.M. (2015). Detecting and visualizing outliers in provider profiling via funnel plots and mixed effect models. Health Care Manag. Sci..

[24] Greenfield S., Kaplan S.H., Kahn R., Ninomiya J., Griffith J.L. (2002). Profiling care provided by different groups of physicians: Effects of patient case-mix (bias) and physician-level clustering on quality assessment results. Ann. Intern. Med..

[25] Garnick D.W., Fowles J., Lawthers A.G., Weiner J.P., Parente S.T., Palmer R.H. (1994). Focus on quality: Profiling physicians’ practice patterns. J. Ambul. Care Manag..

[26] Schectman J.M., Kanwal N.K., Schroth W.S., Elinsky E.G. (1995). The effect of an education and feedback intervention on group-model and network-model health maintenance organization physician prescribing behavior. Med. Care.

[27] Mainous A.G., Hueston W.J., Love M.M., Evans M.E., Finger R. (2000). An evaluation of statewide strategies to reduce antibiotic overuse. Fam. Med..

[28] Balas E.A., Boren S.A., Brown G.D., Ewigman B.G., Mitchell J.A., Perkoff G.T. (1996). Effect of physician profiling on utilization. Meta-analysis of randomized clinical trials. J. Gen. Intern. Med..

[29] McMullin S.T., Lonergan T.P., Rynearson C.S., Doerr T.D., Veregge P.A., Scanlan E.S. (2004). Impact of an evidence-based computerized decision support system on primary care prescription costs. Ann. Fam. Med..

[30] Guthrie B., Kavanagh K., Robertson C., Barnett K., Treweek S., Petrie D., Ritchie L., Bennie M. (2016). Data feedback and behavioural change intervention to improve primary care prescribing safety (EFIPPS): Multicentre, three arm, cluster randomised controlled trial. BMJ Clin. Res..

[31] Mazzaglia G., Caputi A.P., Rossi A., Bettoncelli G., Stefanini G., Ventriglia G., Nardi R., Brignoli O., Cricelli C. (2003). Exploring patient- and doctor-related variables associated with antibiotic prescribing for respiratory infections in primary care. Eur. J. Clin. Pharmacol..

[32] Orzella L., Chini F., Giorgi Rossi P., Borgia P. (2010). Physician and patient characteristics associated with prescriptions and costs of drugs in the Lazio region of Italy. Health Policy.

[33] Berthold N., Kosturkova N., Müller A. (2010). Die Bundesländer im Standortwettbewerb-gestern, heute und morgen [Federal states in competition-yesterday, today, and tomorrow]. ifo Schnelldienst.

[34] Hessisches Ministerium für Wirtschaft, Energie, Verkehr und Landesentwicklung (2014). Bundesländer-, Regional- und Städterankings im Vergleich [Rankings of Federal States, Regions, and Cities—A Comparison].

